# Wound Healing Efficacy of Rosuvastatin Transethosomal Gel, I Optimal Optimization, Histological and In Vivo Evaluation

**DOI:** 10.3390/pharmaceutics14112521

**Published:** 2022-11-19

**Authors:** Randa Mohammed Zaki, Vidya Devanathadesikan Seshadri, Alanoud S. Mutayran, Lara A. Elsawaf, Abubaker M. Hamad, Alanood S. Almurshedi, Rehab Mohammad Yusif, Mayada Said

**Affiliations:** 1 Department of Pharmaceutics, College of Pharmacy, Prince Sattam Bin Abdulaziz University, P.O. Box 173, Al-Kharj 11942, Saudi Arabia; 2 Department of Pharmaceutics and Industrial Pharmacy, Faculty of Pharmacy, Beni-Suef University, Beni-Suef 62514, Egypt; 3 Department of Pharmacology and Toxicology, College of Pharmacy, Prince Sattam Bin Abdulaziz University, P.O. Box 173, Al-Kharj 11942, Saudi Arabia; 4 Department of Pathophysiology, College of Health Sciences, AL-Rayan Colleges, Al-Hijra Road, Madinah Al Munawwarah 42541, Saudi Arabia; 5 Department of Pharmaceutics, College of Pharmacy, King Saud University, P.O. Box 2457, Riyadh 11451, Saudi Arabia; 6 Department of Pharmaceutics, Faculty of Pharmacy, Mansoura University, Mansoura 35516, Egypt; 7 Department of Pharmaceutics and Pharmaceutical Technology, College of Pharmacy, Taibah University, P.O. Box 30039, Madinah Al Munawwarah 41477, Saudi Arabia; 8 Department of Pharmaceutics and Industrial Pharmacy, Faculty of Pharmacy, Cairo University, Cairo 11562, Egypt

**Keywords:** rosuvastatin, wound healing, transethosomes, I optimal design, histology

## Abstract

This study aimed to make a formulation and statistical optimization of transethosomal formulations of rosuvastatin (ROS) to enhance its topical wound healing efficiency. Design-Expert^®^ software was used to employ I optimal design. The formulation variables in the study were surfactant concentration (%*w*/*v*), ethanol concentration (%*w*/*v*) and surfactant type (span 60 or tween 80), while the dependent responses were entrapment efficiency percent (EE%), vesicle size (VS) and zeta potential (ZP). The numerical optimization process employed by the design expert software resulted in an optimum formula composed of 0.819439 (%*w*/*v*) span 60, 40 (%*w*/*v*) ethanol and 100 mg lecithin with a desirability of 0.745. It showed a predicted EE% value of 66.5517 vs. 277.703 nm and a ZP of −33. When it was prepared and validated, it showed less than a 5% deviation from the predicted values. The optimum formula was subjected to further characterizations, such as DSC, XRD, TEM, in vitro release, the effect of aging and wound healing efficiency. The DSC thermogram made a confirmation of the compatibility of ROS with the ingredients used in the formulation. XRD showed the encapsulation of ROS in the transethosomal vesicles. The TEM image pointed out the spherical nature of the nanovesicles with the absence of aggregation. Additionally, the optimum formula revealed an enhancement of drug release in comparison with the drug suspension. It also showed good stability for one month. Furthermore, it revealed good wound healing efficiency when compared with the standard silver sulphadiazine (1% w/w) ointment or the drug-loaded gel, which could be related to the enhanced penetration of the nanosized vesicles of TESMs into the skin, which enhances the wound healing process. So, it could be regarded as a promising carrier of ROS for the treatment of chronic wounds.

## 1. Introduction

Wounds are considered an important risk factor for morbidity all over the world due to microbial infections [1]. Wound healing is a complex process of multiple phases, including homeostasis, inflammatory reactions, cell proliferation and tissue remodeling [2]. The delay in healing the wounds may be due to disturbance in the regular sequence of cellular and biochemical actions, which bring about the re-establishment of the integrity of the skin [3]. Co-existing health disorders (e.g., immunosuppression, diabetes and chronic peripheral vascular disorders) and/or some complications, such as infections and inflammatory conditions, are predisposing factors that lead to delayed wound healing [4]. Therefore, the chronic nature and related complications of wounds due to delayed healing have led to the appearance of nanosized drug delivery systems that aim to assist the healing process of wounds [3]. 

Statins are a class of drugs that are well-known for their lipid-lowering effect; accordingly, they are generally given for the treatment of cardiovascular diseases [5]. Recently, research has shown their ability to manage conditions other than heart problems, such as neurological conditions [6], cancer [7] and inflammation [8], in addition to many dermatological conditions, such as urticaria [9], acne [10], psoriasis [11] and wound healing [12]. Rosuvastatin (ROS) is one of the statins that lowers low-density lipoprotein and raises high-density lipoprotein by inhibiting 3-hydroxy3-methyl glutaryl co-enzyme A (HMG-COA) reductase [13]. It was experimentally proven to be effective in wound healing as it reverses the effect of the inhibitors of wound healing, such as farnesyl pyrophosphate (FPP), and stimulates microvascular and endothelial functions, which enhance wound healing processes [14]. In addition, it interferes with the synthesis of selective proteins in bacteria by blocking many cellular processes and biosynthetic pathways. This increases its capability to stop the formation of key MRSA toxins, which delay the growth of septic skin lesions [15]. In a study by Maged et al., rosuvastatin was loaded in chitosan scaffolds to be applied topically for wound healing which showed enhanced skin healing and regeneration [12]. It was also loaded by Salem et al. into a hydrogel containing nano-cubic vesicles, which were capped with silver nanoparticles for wound healing management, where it showed enhanced wound healing and tissue repair [3]. Unfortunately, ROS suffers from many drawbacks, such as low solubility in water, slow dissolution rate and low bioavailability (less than 20%) [16]. Consequently, the topical route is a good substitute for oral ROS in wound healing management to rise above such drawbacks.

The topical route has many advantages over the oral route in wound healing for many reasons, such as avoiding drug degradation in the liver [3], lowering the systemic side effects [17], easy application, coverage of large surface areas of the body [3], accelerating healing and reduced resistance of bacteria [18].

Nanovesicles (glycerosomes, ethosomes and transferosomes) were successfully applied topically for wound management [19,20]. Liposomes, which are phospholipid bilayer (PC) vesicles having one or more lipid bilayers surrounding an aqueous compartment [21], opened a new research area when used topically for the delivery of triamcinolone [22]. After that, new topical nano-drug delivery systems were developed [23,24,25,26]. Cevc and Blume (1992) introduced transferosomes, which are elastic or deformable liposomes [27] composed of PC bilayer and surfactant. The presence of surfactant molecules in transferosomes made them elastic vesicles, which reduced their rupture, particularly when applied to the skin. They have the ability to deform and pass through a narrow constriction (from 5 to 10 less than the diameter of the vesicles), which enhances skin penetration of the vesicles. The presence of surfactant molecules can cause a disruption of the lipid and protein packing in the stratum corneum [21]. They were proven through many reports to be more effective than rigid liposomes. However, many studies showed that transferosomes were unable to penetrate the stratum corneum lower layers [28]. Then, ethosomes were developed by Touitou et al., which differ from liposomes by having a relatively high concentration of ethanol in the formula [29]. The presence of ethanol increases the fluidity of the subcutaneous lipids [28]. After that, transethosomes (TESMs) were developed, which have the basic components of ethosomes in addition to surfactants or penetration enhancers [30]. So, TESMs have the properties of both transferosomes and ethosomes. They have the ability to cross the skin, reaching the epidermis and dermis [28]. Many surfactants were used for the formulation of transferosomes and ethosomes: life span 80, span 60, sodium cholate, sodium deoxycholate, tween 80, tween 60 and tween 20 [31]. To the best knowledge of the authors, TESM formulations of ROS have not yet been investigated in the literature.

The aim of our study was to formulate ROS in transethosomal gel to explore its capability for topical wound healing.

## 2. Materials and Methods

### 2.1. Materials

Rosuvastatin calcium was gifted by the Aljazeera Company for pharmaceuticals. Lecithin, span 60, tween 80, ethanol and hydroxypropyl methylcellulose (HPMC K4M) were all purchased from Sigma Aldrich (St. Louis, MO, USA).

### 2.2. Statistical Design of ROS Loaded TESMs

Design Expert^®^ software (Ver. 12, Stat-Ease, Minneapolis, Minnesota, USA) was used to implement the study by applying the I optimal design to study the effect of different independent variables on the studied responses. The formulation variables were surfactant concentration (X1), which lay between 0.5 and 1 (%*w*/*v*), ethanol concentration (X2) which ranged from 20 to 40 (%*w*/*v*), and surfactant type, which was either span 60 or tween 80. This produced 19 experimental runs. ROS and lecithin were kept constant in formulations of 20 mg and 100 mg, respectively. The studied responses were entrapment efficiency percent (EE%) (Y1), vesicle size (VS) (Y2) and zeta potential (ZP) (Y3). Table 1 refers to the independent (low and high levels) and dependent variables. Table 2 demonstrates the composition of ROS-loaded TESMs.

### 2.3. Preparation of ROS Transethosomal Formulations

TESMs were prepared by applying the thin film hydration technique [20], where lecithin, ROS and surfactant were dissolved in 10 mL chloroform–methanol mix at a ratio of 2:1 in a round bottom flask, followed by evaporating the organic solvent using a rotary evaporator (Buchi Rotavapor R-200, Switzerland) by applying a vacuum at a temperature of 60 °C at 90 rpm until the formation of a thin film. Then, 10 mL of water containing the calculated amounts of ethanol was used to hydrate the film at 60 °C, which was higher than the transition temperature of the lipid phase (Tc) [28]. 

### 2.4. Evaluation of ROS Transethosomal Formulations

#### 2.4.1. Measurement of Entrapment Efficiency (EE%)

A cooling centrifuge (SIGMA 3–30 K, Sigma, Steinheim, Germany) was used to separate transethosomal vesicles from the un-entrapped ROS by centrifugation at 17,000 rpm for 1 h at 4 °C [32]. Then, a UV spectrophotometer (Shimadzu UV-1800, Kyoto 604-8511, Japan) was used to quantify the ROS concentration in the supernatant after being suitably diluted. The measurements were performed at the predetermined λ_max_ (245 nm). The method was validated in terms of linearity within the concentration, which ranged from 2 to 16 µg/mL (R^2^ of 0.9995).

The EE% was calculated by the use of the following equation [33]:

(1) $$\%\mathrm{EE}=\frac{\mathrm{TD}\;-\;\mathrm{FD}}{\mathrm{TD}}\times 100$$

where EE% is the percent of entrapment efficiency, FD is the amount of free drug, and TD is the amount of the total drug.

#### 2.4.2. Measurement of Vesicle Size (VS), Polydispersity Index (PDI) and Zeta Potential (ZP)

The measurements of the VS, PDI and ZP of the prepared ROS-loaded TESMs were performed using a Zetasizer Nano ZS instrument (Malvern Instruments, Worcestershire, UK) at 25 °C after being suitably diluted with distilled water [33,34]. Each measurement was performed three times.

### 2.5. Statistical Analysis, Optimization and Validation 

The studied responses were subjected to analysis using a factorial analysis of variance (ANOVA) applying Design Expert^®^ software. The optimum formula with the highest EE% and ZP, and the smallest VS was selected using a desirability function. Then, it was prepared and subjected to evaluation in terms of EE%, VS and ZP to verify the validity of the applied statistical models by calculating the percentage relative errors between the predicted values and the measured results by applying the following equation [34,35].

(2) $$\%\;\mathrm{Relative}\;\mathrm{error}=\;\frac{\mathrm{predicted}\;\mathrm{value}\;-\;\mathrm{experimental}\;\mathrm{value}}{\mathrm{predicted}\;\mathrm{value}}\;\times \;100$$

### 2.6. Evaluation of the Optimum ROS Transethosomal Formula

#### 2.6.1. Differential Scanning Calorimetry (DSC)

Pure ROS, a physical mixture of lecithin, span 60, and ROS, and the optimum formula were subjected to a DSC analysis by means of a differential scanning calorimeter (DSC N-650; Scinco, Italy). About 5 mg of each sample were placed in the apparatus’s aluminum pan, followed by heating at a rate of 10 °C per minute until 200 °C underflows of inert nitrogen.

#### 2.6.2. X-ray Diffraction Study (XRD)

Ultima IV Diffractometer (Rigaku Inc. Tokyo, Japan at College of Pharmacy, King Saud University, Riyadh, Saudi Arabia) was used to measure the X-ray diffraction patterns of pure ROS, physical mixture of lecithin, span 60 and ROS, and the optimum formula. They were subjected to scanning at a rate of 10° per minute of speed in the range from 0–60° (2θ). 

#### 2.6.3. Transmission Electron Microscopy (TEM)

The optimum formula morphology was visualized using a transmission electron microscope (TEM; JEOL JEM-1010, Tokyo, Japan). The samples were subjected to suitable dilutions. Then, they were placed on a carbon-coated copper grid. After that, they were coated with 2% *w*/*v* phosphotungstic acid, followed by keeping them in the air for 5 min to be dried. Then, they were imaged using TEM operated under an acceleration voltage of 80 kV [36] and X80000 power of magnification at room temperature.

#### 2.6.4. In Vitro Release

The release of ROS from the optimum ROS-loaded TESMs formula in comparison with the ROS-loaded TESMs gel and drug suspension was studied by introducing the equivalent of 5 mg ROS of each in the dialysis bags, followed by suspending each in a dissolution medium of 250 mL (phosphate buffer pH (7.4)) [37] in the dissolution apparatus (Pharm Test, Hainburg, Germany) at 37 °C and stirring at 100 rpm. Samples of 5 mL were withdrawn from the dissolution media at 1, 2, 3, 4, 5 and 6 h and instantaneously replaced with a fresh medium of an equal volume. After that, the concentration of ROS in the gathered samples was determined using a UV spectrophotometer at 245 nm. The percent of ROS released at different time points was calculated as follows: [38]

(3) $$\mathrm{Qn}=\frac{\mathrm{Cn}\;\times \;\mathrm{Vr}+\sum_{i=1}^{n-1}\mathrm{Ci}\;\times \;\mathrm{Vs}}{\mathrm{initial}\;\mathrm{drug}\;\mathrm{content}}$$

Where

1. Qn: Cumulative percent of ROS released
2. Cn: Concentration of ROS in the dissolution medium at the n^th^ sample
3. Vr: Volume of dissolution medium
4. Vs: Volume of sample
5. $\sum_{i=1}^{n-1}\mathrm{Ci}\sum_{i=1}^{n-1}\mathrm{Ci}$: The summation of the concentrations measured previously

The percentage of ROS released (Q_n_) at various time points was plotted vs. the corresponding time to obtain the release profile of the optimum ROS-loaded transethosomal formula in comparison with the drug suspension.

#### 2.6.5. Effect of Aging

The stability of the optimum ROS-loaded transethosomal formula was determined as a function of time regarding EE%, VS and ZP after placing the formulation in an air-tight vial and keeping it at 4 °C and away from light for one month [39].

### 2.7. Preparation of ROS Transethosomal Gel

The optimum formula was incorporated in a gel base to be applied topically in the in vivo studies. The polymer of choice for the gelling process was hydroxypropyl methylcellulose (HPMC, K4M) at a concentration of 2.5%. Gel preparation was performed by dispersing 0.25 g HPMC in 10 mL distilled water while stirring at 1000 rpm until the formation of a homogenous system. The optimum TESM formula was subjected to ultracentrifugation followed by dispersing the residue (ROS-loaded TESMs) in the gel base to obtain a final formulation with 1% ROS concentration [32].

### 2.8. In Vivo Evaluation of Wound Healing Efficiency

#### 2.8.1. Excision Wound Model

##### Animals

Male Wistar rats weighing 150 ± 20 g were used in the study. The study was approved by the Institutional Animal Ethical Committee (IAEC) (number SCBR-026-2022) of CPCSEA (Committee for Control and Supervision of Experiments on Animals), Prince Sattam Bin Abdulaziz University. They were housed under standard controlled conditions (24 °C and a 12 h light–dark cycle) and provided with a standard rodent pellet diet and water ad libitum.

##### Grouping and Dosing of Animals

Male rats weighing 120 ± 20 g were separated into five groups. Each group contained six animals. The first group contained control animals (normal animals without wound induction). The second group contained animals with a wound who did not receive any treatment. The third group took standard silver sulphadiazine (1% *w/w*) ointment as a treatment. The fourth group received drug-loaded gel (1% ROS in 2.5% HPMC, K4M). Finally, the last group received the optimum ROS transehosomal gel formula. Before the start of the study, the animals were supplied with standard food and water ad libitum and acclimatized to the laboratory conditions. 

##### Experimental Design

The creation and excision of wounds on the rats were initiated by making anesthesia using an IV injection of ketamine (120 mg/kg body weight) followed by shaving the mice’s backs. Then a scalpel and sharp scissors were used to create the wound on the sides of the central trunk, followed by sterilization using ethanol and removing the skin from the marked area to obtain a wound of 135 mm^2^ at maximum. Afterward, wound cleaning was performed using a cotton swab soaked in saline, followed by the placing of the animals in individual cages and the gentle application of the different treatments 24 h after wound induction once per day by covering the wound until complete healing. A transparent ruler was used to measure each animal’s wound diameter at 0, 7, 14 and 21 days on a weekly basis until epithelialization and the recording of complete wound closure. The wound area gave an indication of the activity of wound healing, in addition to the wound contraction rate percent [40]. The following equation was used to calculate the percent wound contraction:

(4) $$\%\mathrm{wound}\;\mathrm{contraction}=\frac{\;\mathrm{Initial}\;\mathrm{size}\;\mathrm{of}\;\mathrm{the}\;\mathrm{wound}\;-\;\mathrm{Wound}\;\mathrm{size}\;\mathrm{in}\;a\;\mathrm{specific}\;\mathrm{day}\;}{\mathrm{Initial}\;\mathrm{size}\;\mathrm{of}\;\mathrm{the}\;\mathrm{wound}}\times 100$$

Samples of skin tissue (3–5 cm) from different animal groups were instantly dipped in a suitable amount of 10% formalin.

Wound healing models cause moderate to severe pain. Multimodal strategies and therapy paved the way for modern robotic surgeries to take place so as to reduce the need for frequent doses of painkillers, faster recovery and the complete healing of wounds to prevent the nightmare of chronic pain. This became the key interest in our study: to look out for a speedy recovery in all ways from the new drug [41].

#### 2.8.2. Histological Study

To compare the histological effect of the investigated material on wound healing, we used two stains on formalin-fixed paraffin wax as a fixed representative and a suitable size of skin tissue biopsies. Thus, representative wound skin tissue samples with a thickness from 3–5 cm from the five animal groups were instantly immersed in a suitable amount of 10% formalin and prepared in an automatic tissue processing machine (ASP300s, Leica Bio systems, Buffalo Grove, IL, USA), followed by impeding them in paraffin wax blocks. Then 5 µ thick sections were prepared using a rotary microtome (SHUR/Cut 4500, TBS, Durham, NC, USA) [42]. Two sections of each block were taken for staining; one was stained with the hematoxylin and eosin (H&E) technique for general tissue appearance staining, and the second was stained with the Masson trichrome technique (MT) for connective tissue fibers, mainly collagen, which takes blue color [43,44]. The hematoxylin and eosin method was performed by the following descriptions of Bancroft and Layton [44]. The Masson trichrome techniques were completed according to Hamad et al. [43].

## 3. Results and Discussion

### 3.1. Evaluation of ROS Transethosomal Formulations

#### 3.1.1. Measurement of EE%

**Figure 1:**
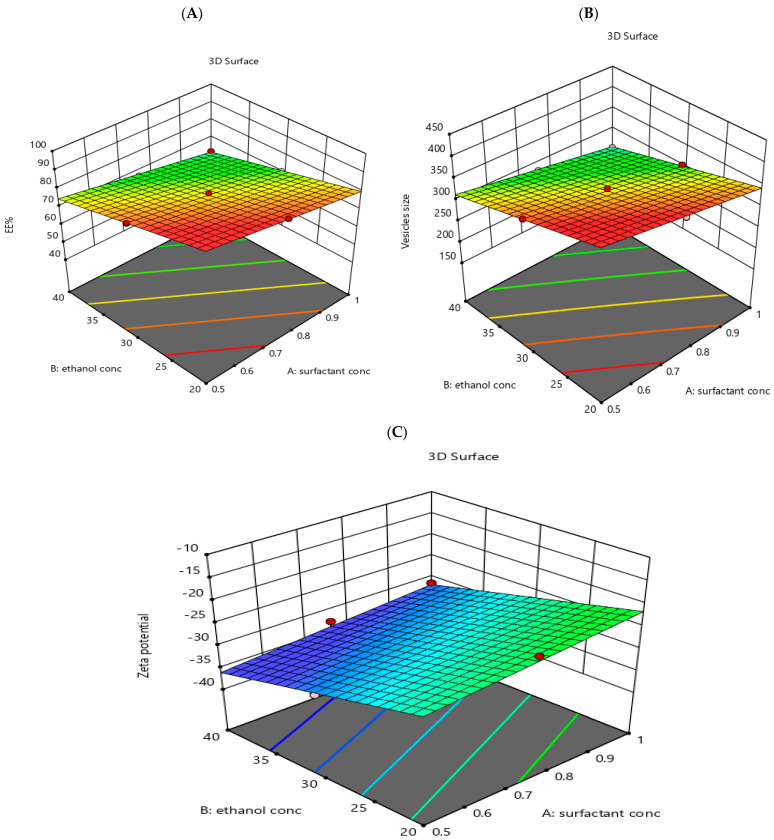
3D response surface plot for the effect of independent variables: surfactant concentration (**A**), ethanol concentration (**B**) and surfactant type on the dependent responses, EE% (**A**), vesicle size (**B**), and Zeta potential (**C**).

**Figure 2:**
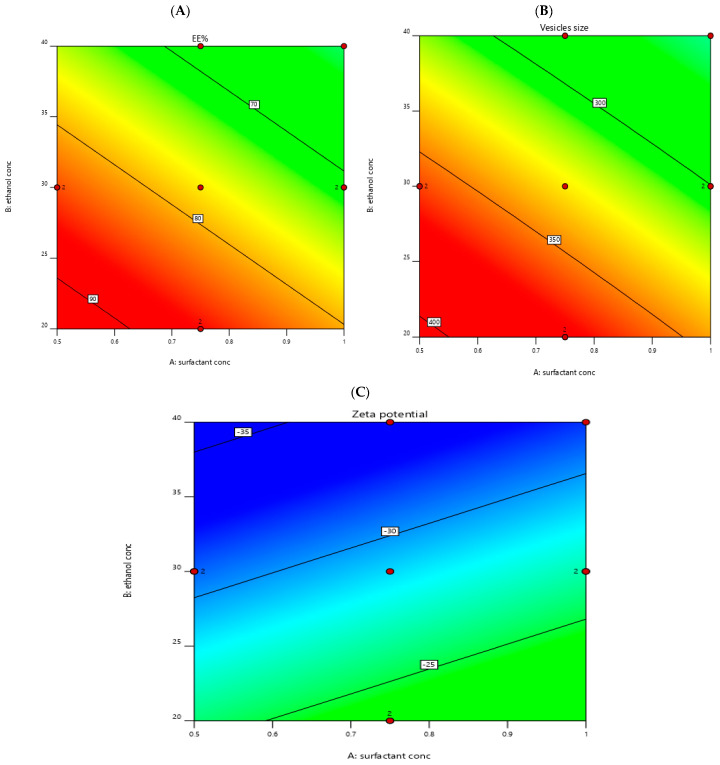
Contour plot for the effect of independent factors on different responses, EE% (**A**), vesicle size (**B**), and Zeta potential (**C**).

The EE% of the prepared transethosomal formulations varied between 45.2 ± 2.58 and 87.3 ± 3.62, as shown in Table 2. The effects of surfactant concentration %*w*/*v* (X1) and ethanol concentration %*v/v* (X2) on EE% are shown in Figure 1A and Figure 2A.

The linear model was the most suitable one fitted to EE% data (*p*-value < 0.0001), where the lack of fit is non-significant (*p*-value 0.132), and the difference between the adjusted and predicted R^2^ was small (less than 0.2), which points out that the model is valid [34]. The adequate precision was high, 88.1062 (greater than four), as shown in Table 3. This referred to the ability of the model to navigate the design space [45,46]. 

The effect of the independent variables on EE% was shown in the following equation:

EE% = +68.96 − 6.51 X1 − 9.22 X2 − 8.62 X3(5)

It can be concluded from the ANOVA analysis shown in Table 4 that all three independent variables namely, surfactant concentration %*w*/*v* (X1), ethanol concentration %*v/v* (X2) and surfactant type, have a significant effect on EE% values with *p*-values < 0.0001.

Increasing both surfactant and ethanol concentrations led to a significant decrease in EE%, as indicated by the negative sign of their coefficients in the correlation equation.

Regarding the effect of surfactant concentration on EE%, the decrease in EE% with the increase in surfactant concentration could be related to increasing the membrane permeability of the vesicles, which resulted from the arrangement of the surfactant molecules within the lipid bilayer structure of the vesicles, which led to the generation of pores, within the membrane resulting in increasing its fluidity which increased leakage of the entrapped drug [47]. 

Concerning the effect of ethanol concentration on EE%, there was a decrease in the EE% with the increase in the concentration of ethanol, which could be explained by the solubilization of the drug in ethanol in addition to the effect of ethanol on the vesicle’s membrane, which led to enhanced fluidity and permeability with the subsequent loss of the drug from it [48]. Our findings complied with those published by Abdulbaqi et al. [49].

For the effect of surfactant type on EE%, it was obvious that the EE% was higher in formulations containing span 60 in comparison w those prepared with tween 80. These results were in agreement with the results published by Aboud et al. [50] and could be referred to as the hydrophilic-lipophilic balance (HLB) values of span 60 and tween 80, which were 4.7 and 15, respectively [28,50,51]. Surfactants with low HLB are lipophilic and would prefer the entrapment of lipophilic drugs [52]. This explained the increased EE% of ROS, which is a lipophilic drug [16], in formulations containing span 60 than those containing tween 80. In addition, there was some kind of interaction between the hydrophobic alkyl chain of span 60 and the hydrophobic domain in the vesicles, which results in more condensed layers and so prevents the leeching of drugs from the vesicles [53]. Contrarily, surfactants with high HLB, such as tween 80, are more hydrophilic and form vesicles with less rigid membranes, which is related to the larger polar head groups in addition to increasing the solubilization of the drug in the aqueous medium during preparation, which led to lowering the EE% of ROS [54].

#### 3.1.2. Measurement of VS, PDI and ZP

The VS of the prepared transethosomal formulations lay between 191.4 ± 7.84 to 372.6 ± 12.84 nm, as shown in Table 2. The effects of the surfactant concentration *w*/*v*% (X1) and ethanol concentration *v/v*% (X2) on VS are shown in Figure 1B and Figure 2B.

The most appropriate model to be fitted to VS data was the two-factor interaction (*p*-value < 0.0001) with a non-significant lack of fit (*p*-value 0.154) and a small difference between the adjusted and predicted R^2^ (less than 0.2), which ensures the validity of the model [34]. The adequate precision was high, 65.0316 (greater than 4); this pointed out the ability of the model to navigate the design space [45,46], as shown in Table 3. 

The effect of the studied factors on VS was refereed in the following equation:

VS = +284.47 − 27.41 X1 − 37.25 X2 − 46.01 X3 + 1.07 X1X2 + 2.59 X1X3 + 7.49 X2X3(6)

It was obvious from ANOVA analysis, as seen in Table 4, that surfactant concentration *w*/*v%* (X1), ethanol concentration *v/v*% (X2) and surfactant type all have a significant effect on VS values with (*p*-values < 0.0001).

Increasing both surfactant and ethanol concentrations resulted in a significant decrease in VS, as shown by the negative sign of their coefficients in the correlation equation. The decrease in VS with the increase in surfactant concentration could be related to the softening of the membrane and increased elasticity, which results in an increased reduction ability [55]. Our results were in agreement with the results obtained by Chen et al. [56]. It was also noted from the results that the PS was in accordance with the amount of drug entrapped within the vesicles and explained that decreasing the EE% of the vesicles led to reducing its size [28].

The decrease in VS with the increase in the concentration of ethanol could be attributed to the reduction in the thickness of the membrane and due to the formation of a phase with interpenetrating hydrocarbon chains [57]. The presence of ethanol gave steric stabilization to vesicles due to imparting some negative charge, which results in enhancing the physical stability of particles and preventing their aggregation [58]. Our results met the results published by Nayak et al. [59]. 

Concerning the effect of surfactant type on VS, all formulae prepared using Span 60 are larger in size than those prepared using Tween 80. This could be related to the HLB values of each surfactant. Yeo et al. [60] pointed out that when the HLB value of a surfactant decreases, the VS increases, which could be linked to the reduction of the hydrophilic portion of the surfactant. That is why span 60 with the lower HLP value (4.7), as mentioned before, showed a higher VS than tween 80 with an HLP value of 15. Our results complied with results published by Rofida et al. [28].

PDI shows the variety in size between particles and is referred to by values between 0 and 1 [45]. As presented in Table 1, the PDI values of the prepared transethosomal formulations varied between 0.137 ± 0.033 and 0.501 ± 0.148; this indicated the acceptable size distribution for the prepared transethosomal formulations [45]. 

ZP points out the physical stability of the prepared transethosomal formulations. Where increasing the ZP value leads to increasing the repulsion forces between vesicles, which reduces their aggregation and increases system stability [61]. 

As seen in Table 2, the ZP of the prepared transethosomal formulations lay between −14.3 ± 2.64 and −32.7 ± 1.38 mV. This refers to the physical stability of the prepared transethosomal formulations [62]. The effects of surfactant concentration (X1) and ethanol concentration (X2) on ZP are shown in Figure 1C and Figure 2C.

The most appropriate model to be fitted to the ZP data was the linear model (*p*-values < 0.0001). The adequate precision was high (46.9420), and the difference between the adjusted and predicted R^2^ was less than 0.2. The effect of the studied factors on ZP could be clarified using the proceeding equation:

ZP = −24.94 + 2.13 X1 + 5.12 X2 + 3.83 X3(7)

The ANOVA analysis in Table 3 shows that both surfactant concentration (X1), ethanol concentration (X2) and surfactant type (X3) significantly affected ZP (*p*-values < 0.0001), where X1 and X2 significantly increased ZP absolute values. The increase of the ZP absolute value with the increase of the surfactant concentration could be related to the charge imparted by them on the vesicles’ surface [47]. The increase in the ZP absolute value with the increase in the ethanol concentration could be referred to as imparting a negative charge on the vesicles^’^ surface, which led to electrostatic repulsion between them, avoiding their aggregation [49]. Our results were in agreement with that published by Dayan and Touitou [63].

Regarding the effect of surfactant type on ZP values, the ZP of the formulations prepared using tween 80 is less than that of those prepared using span 60, which could be explained by the higher HLP values of tween 80 in comparison with span 60. Our results complied with those published by Rofida et al. [28]. Kim et al. [64] pointed out that the HLP value of the surfactant affects the competitive adsorption of OH ions present in the hydration medium at the interface. The lower the HLP value of the surfactant, the higher the adsorption of OH ions on the nonpolar interface and the higher the ZP. The presence of (CH_2_-CH_2_-O)_n_ in tween 80 made hydrogen bonds with water molecules, which led to the lowering of the ZP value [65].

### 3.2. Statistical Analysis, Optimization and Validation 

**Figure 3:**
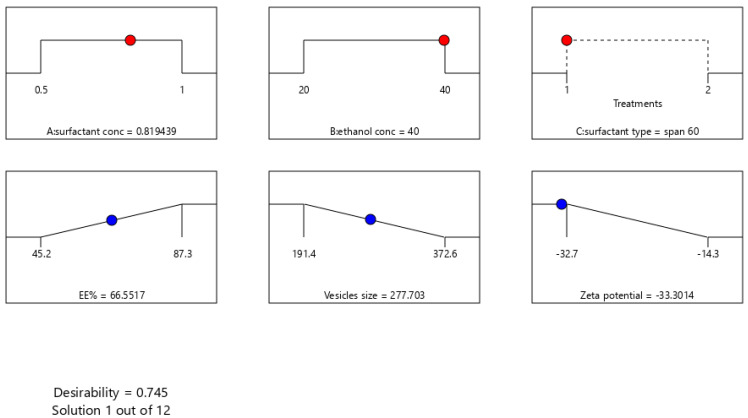
The composition of the optimized formula with its expected responses according to I optimal design.

**Figure 4:**
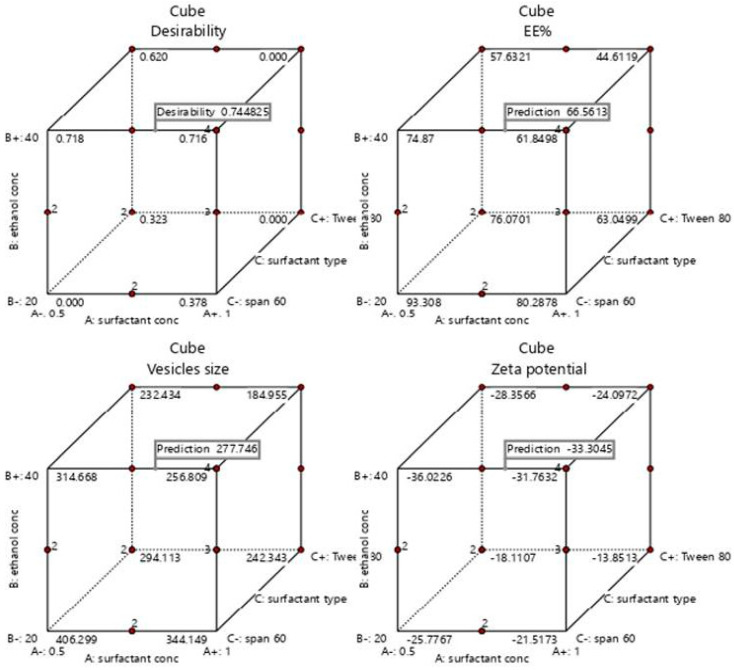
Cube graph for the expected responses of the optimized formula with its desirability.

A numeric analysis for the selection of the optimum transethosomal formula was made by applying Design Expert^®^ software, where EE% and ZP were maximized while VS was minimized. This resulted in an optimum transethosomal formula with a desirability of 0.745 (Figure 3). Its composition was 0.819439 (*w*/*v%*) span 60, 40 (*w*/*v%*) ethanol and 100 mg lecithin. The predicted values of EE%, VS and ZP were 66.5517%, 277.703 nm and −33.3014 mV, respectively, as shown in Table 5 and Figure 3 and Figure 4. The optimum formula was prepared and then validated, as verified in Table 4, with a percentage of relative errors of less than 5% from the predicted values produced by the design expert software, which indicated the fitness of the model [35].

### 3.3. Evaluation of the Optimum ROS Transethosomal Formula

#### 3.3.1. Differential Scanning Calorimetry (DSC)

**Figure 5:**
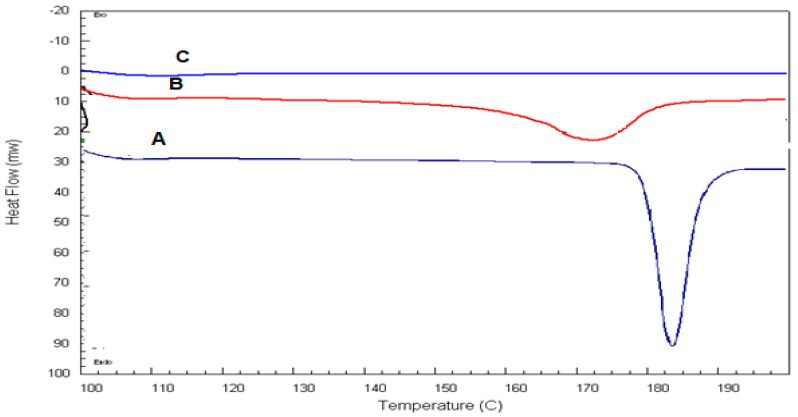
DSC thermograms of (**A**): pure ROS, (**B**): lecithin, span 60, and ROS physical mixture, (**C**): the optimum formula.

DSC thermograms of pure ROS, physical mixture of lecithin, span 60 and ROS and the optimum transethosomal formula are shown in Figure 5. Pure ROS exhibited an endothermic peak at 184 °C, which indicated its melting point in crystal form (Figure 5A) [66,67]. The endothermic peak of the drug was well conserved in its physical mixture with lecithin and span 60 (Figure 5B), with changes in the form of broadening or shifting the melt temperature. The used quantity of materials, especially in the mixtures of drugs and excipients, may have an effect on the enthalpy and shape of the peak. These minute changes in the melting endotherm of the drug may be due to making a mix between the drug and the excipients, which resulted in reducing the purity of the mixture’s individual components, and this may not essentially point out a probable incompatibility [68,69,70]. In addition, drug crystallinity changes may result in shifts in the melting point [68]. So, the compatibility of ROS with the formulation excipients could be deduced. The optimum transethosomal formula (Figure 5C) showed the absence of the drug’s endothermic peak, which indicated that the drug was encapsulated and converted into an amorphous form [71]. 

#### 3.3.2. X-ray Diffraction Study (XRD)

**Figure 6:**
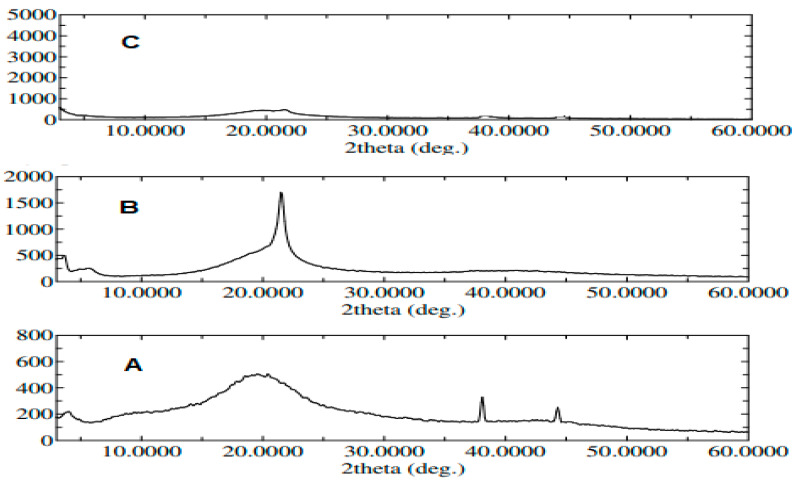
XRD of (**A**): pure ROS, (**B**): lecithin, span 60 and ROS physical mixture, and (**C**): the optimized formula.

The XRD spectra of pure ROS, lecithin, span 60, ROS physical mixture and the optimum formula are shown in (Figure 6). The XRD of pure ROS revealed a broad peak at an angle of 20° and sharp peaks at angles of 38° and 44°, which indicated its crystalline nature [3,12] (Figure 6A). The XRD spectra of lecithin, span 60 and ROS physical mixture showed the appearance of a new sharp peak at an angle of 22° with the persistence of the drug peak at 20° (Figure 6B). However, a decrease in the intensity of the drug peaks was observed in the XRD spectrum of the optimized formula (Figure 6C), which may be due to the drug encapsulation within TESMs nanovesicles in an amorphous form. The obtained findings were in compliance with the DSC results [34]. 

#### 3.3.3. Transmission Electron Microscopy (TEM)

**Figure 7:**
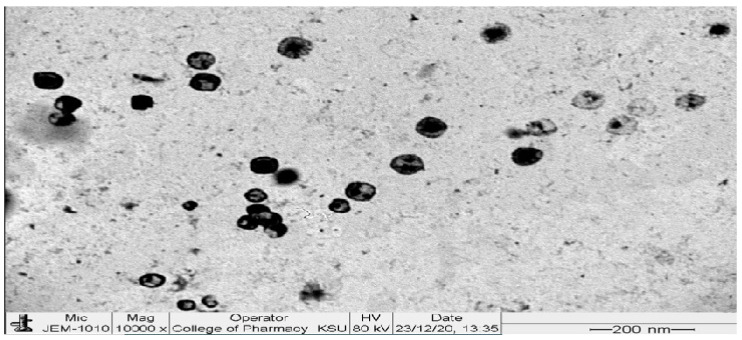
TEM image of the optimized formula.

Photographs of TEM revealed small vesicles of a spherical nature, as seen in Figure 7. No aggregation was observed, which points out that the dispersion was physically stable, and this might be due to the high surface ZP of the TESMs nanovesicles surfaces, which imparts repulsion forces between them [33,34].

#### 3.3.4. In Vitro Release

**Figure 8:**
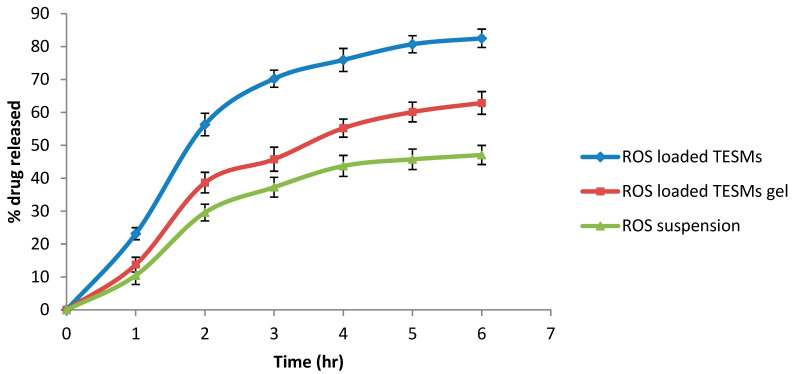
In vitro release profile of ROS from ROS-loaded TESMs compared with ROS-loaded TESMs gel and ROS suspension.

The release profile of the optimum ROS-loaded TESMs formula compared with ROS-loaded TESMs gel and ROS suspension was presented in Figure 8. There was an enhanced release of ROS from ROS-loaded TESMs compared with the drug suspension. This might be referred to as the amphiphilic properties of lecithin used in TESMs formation [72,73]. The reduction in vesicle size of the transethosomal formulation may lead to enhanced drug release [34]. Vesicle size had an effect on the release of the drug from nanovesicles, where smaller vesicles led to a higher release rate in comparison to larger-sized ones [73,74]. Additionally, ROS-loaded TESMs gel showed a slower release rate than ROS-loaded TESMs; this could be attributed to the release from transethosomal nanovesicles and diffusion of ROS through the network structures of the gel, resulting in a controlled release model for ROS-loaded TESMs gel. This result is in agreement with Zaki et al. [32].

#### 3.3.5. Effect of Aging

**Figure 9:**
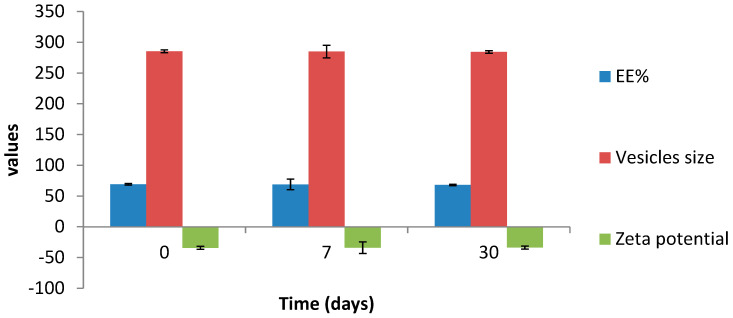
The effect of storage at 4 °C for one month on EE%, vesicle size and zeta potential of the optimized formula.

The effect of one month’s storage on the stability of the optimum transethosomal formula is shown in Table 6 and Figure 9. The EE%, VS and ZP did not significantly change during the study periods (7 and 30 days), which could reflect the good stability of the optimum transethosomal formula during one month’s storage at 4 °C [34].

### 3.4. In Vivo Evaluation of Wound Healing Efficiency

#### 3.4.1. Quantification of Wound Area

**Figure 10:**
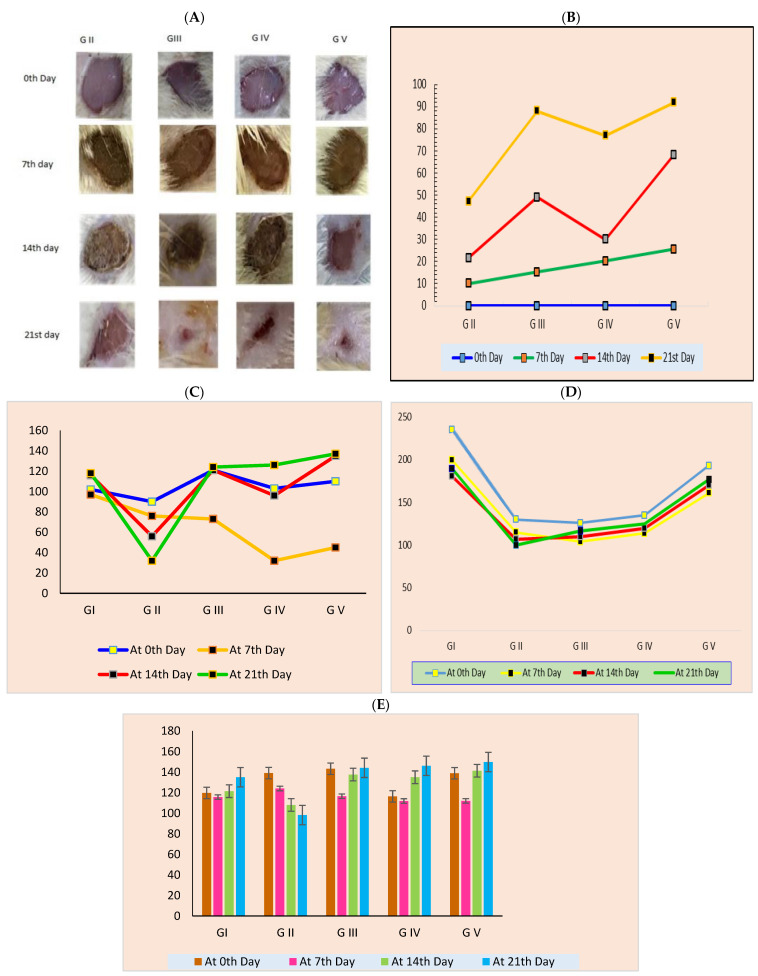
Photographs of wound healing process at different time intervals for different groups (**A**,**B**), effect of drugs on food intake of normal and wound induction rates (**C**), effect of drugs on water intake of normal and wound induction rates (**D**), effect of drugs on body weights of normal and wound induction rates (**E**).

Wound closure was confirmed by measuring the diameter of the wounds. The group that did not receive any treatment (group 2) was used to verify the normal healing activity in the animal model. All animal groups revealed a decrease in wound area daily until the end of the study after 21 days as compared with the start date, as shown in Table 7 and Figure 10A,B. Moreover, wound epithelization time was longer in group two compared with the other treatment groups. As shown in Figure 10A,B, the group five animals, which were treated with the ROS tranethosomal gel formula, showed larger wound closure in comparison with the other groups, which could be related to the penetration of nanosized vesicles of TESMs into different skin layers, which enhances the wound healing process [18,75]. Additionally, the presence of edge activators in the composition of transethosomes enhances skin permeation by increasing the fluidity of transethosomal lipid bilayer and consequently easifying their squeezing into the skin pores [76,77]. Moreover, the high concentration of ethanol enhances skin permeation by two mechanisms: first, it interacts with lipid molecules of stratum corneum causing a change in the packing of skin lipids and consequently increasing their fluidity and permeability; second, it increases the fluidity and flexibility of transethosomal lipid bilayers and so increases their permeation through the skin [28]. Two-way ANOVA analysis showed that all the groups are significantly different from each other in wound healing activity (*p*-value < 0.021254), and also there is a significant difference in wound healing activity on days 7, 14 and 21 (*p*-value < 0.000564), as shown in Table 7. The decrease in wound size in treatment groups compared with the untreated group is shown in Figure 10A,B.

#### 3.4.2. The Effect of Wound Induction and Healing on Body Weight and Food and Water Intake

Figure 10C,D present the food and water intake of the rats. The daily food and water intake of the rats was significantly decreased in all the groups compared with normal animals. Food and water consumption have a direct relation to the health condition of rats; in this case, due to the wound, the animals were unable to move, resulting in a decrease in food and water consumption. The progression of wound healing is indicated by the increase in food and water intake, which is seen in the figures. 

Figure 10E shows the body weight changes in the groups after wound induction and wound healing in the groups. The declining pattern of body weight and regaining of body weight directly attributes to the feeding patterns of the animals. The results clearly show that there are significant body weight changes during the wound 0–7 days of wound induction and later. (14–21 days). Changes in body weight are used to assess the course of the disease and response to drug therapy. Body weight is a good indicator of pain, inflammation and stress that occurs during an injury or wound. Weight loss was observed in all the wound-induced groups, which clearly stated the alleviation of pain, inflammation and stress in the animals. Body weight changes are an important tool for indicating the feeding behavior of the animals, which has been used for a long time to quantify the chronic pain status of various animals [78,79,80].

#### 3.4.3. Histological Study

**Figure 11:**
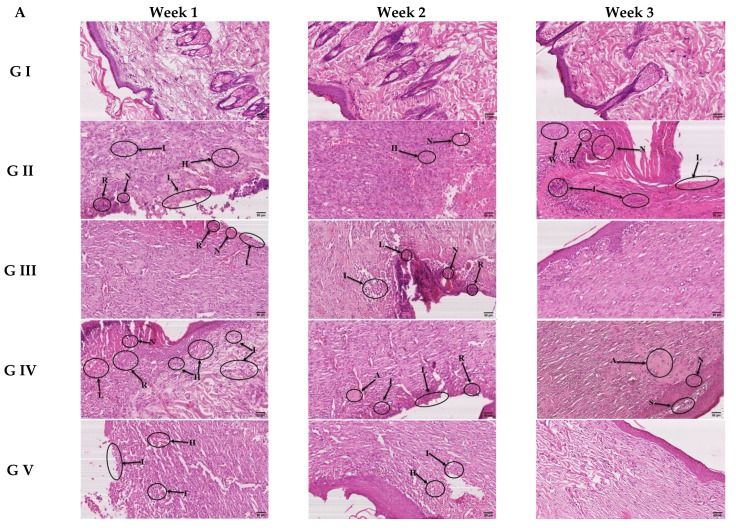

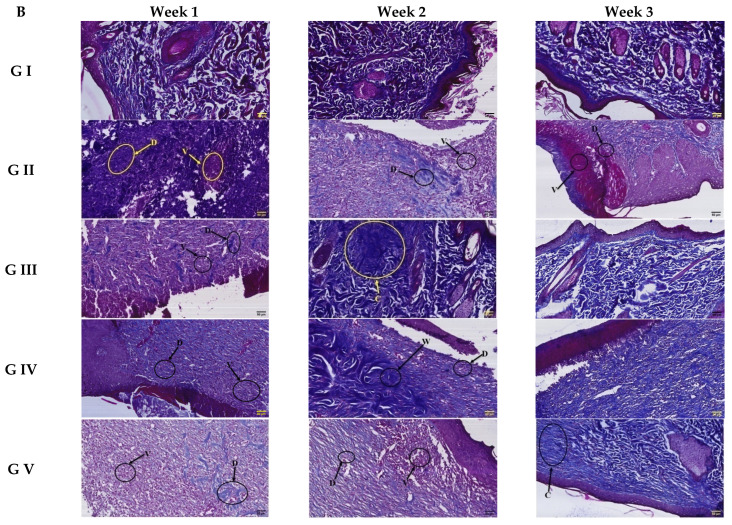
Photographs of H&E-stained sections (**A**) and Masson trichrome (MT) for connective tissue fibers (**B**). L = loss of epithelial tissue layer area, R = epithelialization area, W = normal epithelial tissue area, N = Necrotic tissue area, I = infiltration of inflammatory cells, A = accumulation of hyaline material, S = Separation area remaining (H&E, 200×, Bar = 50 μm), D = decreased amount of collagen fibers, V = very much decreased amount of collagen fibers, C = collagen fibers reformation after wound healing, W = normal appearance of collagen fibers, (MT, 200×, Bar = 50 μm).

Histopathological examination is another piece of evidence for the experimental wound healing activity (Figure 11). Like our study, Zhang et al. [81], in their study of skin wounds, used both methods of H&E as well as the method of Masson trichrome to assess the efficacy of the treatment they applied. Aneesha et al. [82] used the same two histological stains to assess the wound healing of diabetic tissue samples. In addition, histopathological features were assessed for the improvement of skin tissue wound healing by using H&E and Masson trichrome in our study, like in Wahedi et al. [83].

As expected, the control group (group 1), in all three weeks, gave a normal histological appearance of the skin in both H&E stained sections (Figure 11A) as well as MT-stained sections (Figure 11B). Second, the toxic-induced group (group 2) showed very little improvement over the three weeks but suffered from several pathological events such as loss of epithelial tissue layer area (L), necrotic tissue area (N), hemorrhage (H), and infiltration of inflammatory cells (I). MT-stained sections of this group showed continuous suffering of the skin tissue from the decreased amount (D) of collagen fibers in connective tissue near the wound areas while a very much (V) decreased amount of collagen fibers in areas nearer to the wound. Third, the standard treated group (group 3) showed gradual improvement throughout the period of three weeks and almost normal skin tissue appearance in both H&E and MT-stained sections. Fourth, the drug-loaded gel-treated group (group 4) showed high improvement in regard to wound healing, but the tissue of the skin was not completely healed. Finally, the optimum transethosomal gel formula-treated group (group 5) showed better and highly improved skin tissue that showed complete healing and almost normal tissue appearance in both H&E and MT staining, which could be related to the reason previously discussed in Section 3.4.1. In conclusion, this histopathological experiment revealed that the optimum transethosomal gel formula-treated group exhibited different biological behavior in closing the wound area first during the first two weeks and then rapidly continued the healing of connective tissues underneath until the wound was almost normal by the end of the third week.

## 4. Conclusions

In the current study, I optimal design was employed for the optimization of ROS TESMs where EE% and ZP were maximized while VS was minimized. This resulted in an optimum formula composed of 0.819439 (%*w*/*v*) span 60, 40 (%*w*/*v*) ethanol and 100 mg lecithin with a desirability of 0.745. It showed a reasonable vesicle size of 277.703 nm, ZP of −33 and ROS entrapment efficiency of 66.5517%. This optimum formula showed spherical vesicles under TEM with no aggregates, which were confirmed by the stability study for one month. It also showed enhanced drug release when compared with the drug suspension. In addition, DSC and XRD studies showed good compatibility of the drug with the excipients in the formula and revealed its encapsulation within the nanovesicles. Finally, it was subjected to a wound healing efficiency study applying an excision wound model and histology study where it showed good wound healing properties when compared with the standard silver sulphadiazine (1% w/w) ointment, and this could be related to the penetration of the nanosized vesicles of TESMs into the skin, which enhanced the wound healing process. So, it could be regarded as a promising carrier for chronic wound treatment.

## Figures and Tables

**Table 1:** I optimal design for optimization of ROS-loaded TESMs.

Independent Variables	Levels	
	High	Low
Surfactant concentration %*w*/*v* (X1)	0.5	1
Ethanol concentration %*v/v* (X2)	20	40
Surfactant type (X3)	Span 60	Tween 80
**Dependent values (Responses)**	**Desirability**	
EE% (Y1)	Maximize	
Vesicle size (Y2)	Minimize	
Zeta potential (Y3)	Maximize	

**Table 2:** Composition of different formulations with their responses in I optimal design for optimization of ROS-loaded TESMs.

Formula Code	Independent Variables			Dependent Variables			
	Surfactant Concentration *w*/*v%* (X1)	Ethanol Concentration *v/v*% (X2)	Surfactant Type (X3)	EE% (Y1)	Vesicle Size (nm) (Y2)	Zeta Potential (mv) (Y3)	PDI
1	0.75	30	Tween 80	60.3 ± 2.46	237.2 ± 7.45	−20.5 ± 2.63	0.234 ± 0.056
2	1	30	Tween 80	51.5 ± 1.77	202.6 ± 10.73	−18.1 ± 1.36	0.275 ± 0.123
3	0.75	30	Tween 80	60.1 ± 3.11	236.7 ± 5.46	−20.3 ± 1.84	0.198 ± 0.062
4	1	40	span 60	62.3 ± 2.89	254.3 ± 6.34	−31.4 ± 3.52	0.137 ± 0.033
5	0.75	20	span 60	87.3 ± 3.62	372.6 ± 12.84	−22.6 ± 1.74	0.318 ± 0.042
6	1	20	Tween 80	64.5 ± 1.75	246.7 ± 10.46	−14.3 ± 2.64	0.234 ± 0.117
7	0.5	20	Tween 80	74.6 ± 3.28	294.2 ± 9.12	−19.1 ± 3.28	0.272 ± 0.123
8	1	30	span 60	70.7 ± 2.64	302.8 ± 11.87	−27.5 ± 2.98	0.311 ± 0.093
9	1	40	Tween 80	45.2 ± 2.58	191.4 ± 7.84	−25.2 ± 2.57	0.212 ± 0.085
10	0.75	20	span 60	87.1 ± 1.75	372.2 ± 13.56	−22.5 ± 1.38	0.376 ± 0.128
11	0.5	30	span 60	84.2 ± 2.91	361.6 ± 15.43	−31.4 ± 2.91	0.445 ± 0.093
12	1	30	span 60	70.2 ± 2.75	302.4 ± 12.54	−27.3 ± 2.68	0.324 ± 0.121
13	0.75	30	Tween 80	59.8 ± 3.28	236.5 ± 14.21	−20.1 ± 3.01	0.456 ± 0.182
14	0.5	40	Tween 80	58.1 ± 3.27	232.1 ± 10.36	−28.4 ± 2.21	0.385 ± 0.089
15	0.75	30	span 60	78.6 ± 4.25	335.8 ± 6.82	−30.2 ± 1.84	0.172 ± 0.102
16	0.5	30	span 60	83.9 ± 2.78	361.1 ± 18.53	−31.2 ± 2.49	0.501 ± 0.148
17	0.75	40	span 60	67.4 ± 1.96	282.6 ± 13.67	−32.7 ± 1.38	0.438 ± 0.113
18	0.75	40	Tween 80	52.3 ± 2.56	210.6 ± 7.29	−26.4 ± 2.76	0.275 ± 0.138
19	0.75	20	Tween 80	70.5 ± 3.27	271.8 ± 11.33	−16.5 ± 3.72	0.356 ± 0.186

**Table 3:** Output data of I optimal design of ROS-loaded TESMs.

Dependent Variables	R2	Adjusted R2	Predicted R2	Adequate Precision
Y1: %EE	0.9942	0.9930	0.9902	88.1062
Y2: Vesicle size (nm)	0.9956	0.9934	0.9854	65.0316
Y3: Zeta potential (mV)	0.9774	0.9729	0.9648	46.9420

**Table 4:** ANOVA for I optimal design of ROS-loaded TESMs.

Dependent Variable	Source	SS	df	Mean Square	F Value	*p* Value
Y1	Model	2780.54	3	926.85	851.12	<0.0001
	X1	410.63	1	410.63	377.08	<0.0001
	X2	841.22	1	841.22	772.49	<0.0001
	X3	1407.44	1	1407.44	1292.45	<0.0001
Y2	Model	62,751.75	6	10,458.63	450.13	<0.0001
	X1	7089.42	1	7089.42	305.12	<0.0001
	X2	12,607.30	1	12,607.30	542.60	<0.0001
	X3	38,872.69	1	38,872.69	1673.03	<0.0001
	X1X2	5.24	1	5.24	0.2254	0.6435
	X1X3	63.56	1	63.56	2.74	0.1241
	X2X3	509.32	1	509.32	21.92	0.0005
Y3	Model	561.68	3	187.23	216.24	<0.0001
	X1	43.94	1	43.94	50.75	<0.0001
	X2	259.77	1	259.77	300.02	<0.0001
	X3	278.36	1	278.36	321.49	<0.0001

Y1: %EE, Y2: Vesicle size (nm), Y3: Zeta potential (mV), X1: Surfactant concentration *w*/*v%*, X2: Ethanol concentration *v/v*%, X3: Surfactant type, SS: sum of squares, df: degree of freedom.

**Table 5:** The composition and validation of the optimized formula with its predicted responses according to I optimal design.

The Optimized Formula	Independent Variables			Predicted Responses			Desirability
	Surfactant concentration *w*/*v%* (X1)	Ethanol concentration %*v/v* (X2)	Surfactant type (X3)	EE%	Vesicle size	Zeta potential	
	0.819439	40	Span 60	66.5517	277.703	−33.3014	0.745
**Validation of the optimum formula**							
**Responses**	**Predicted value**		**Experimental value**		**% Relative error**		
EE%	66.552		69.142		3.892		
Vesicle size	277.703		285.451		2.79		
Zeta potential	−33.301		−34.27		2.909		

**Table 6:** The effect of storage at 4 °C for one month on EE%, Vesicle size and Zeta potential of the optimized formula.

Responses	Fresh	After 7 Days	After 30 Days
**EE%**	69.142 ± 1.35	68.83 ± 2.18	68.01 ± 2.46
**Vesicle size**	285.451 ± 8.64	284.95 ± 10.23	284.23 ± 9.39
**Zeta potential**	−34.27 ± 1.23	−34.01 ± 2.03	−33.78 ± 2.54

**Table 7:** Percentage of wound healing activity and ANOVA analysis.

% Wound Healing						
	0th Day	7th Day		14th Day	21st Day	
**G II**	0	10		21.6	47.3	
**G III**	0	15.3		49	88	
**G IV**	0	20.3		30	77	
**G V**	0	25.6		68.3	92	
**Two way ANOVA**						
**Source of Variation**	**SS**	**df**	**MS**	**F**	***p*-value**	**F crit**
**Rows**	1929.549	3	643.1831	7.094344	0.021254	4.757063
**Columns**	6041.045	2	3020.523	33.31653	0.000564	5.143253
**Error**	543.9683	6	90.66139			
**Total**	8514.563	11				

## Data Availability

The data is contained in the manuscript.
